# The Singapore Experience With Uncontrolled Gout: Unmet Needs in the Management of Patients

**DOI:** 10.7759/cureus.36682

**Published:** 2023-03-25

**Authors:** Zheng Cong Lee, Anindita Santosa, Andrew Yu Keat Khor, Melonie K Sriranganathan

**Affiliations:** 1 Rheumatology, Changi General Hospital, Singapore, SGP

**Keywords:** treatment challenges, chronic kidney disease (ckd), ischaemic heart disease, unmet need, gout disease

## Abstract

Gout is the most common type of inflammatory arthritis, and its impact on cardiovascular health and quality of life is often underestimated. The prevalence and incidence of gout are increasing globally. Further, ischemic heart disease (IHD) and chronic kidney disease (CKD) are prevalent in gout patients. Some unmet needs for gout management include physicians' low initiation rate of urate-lowering therapy (ULT) and poor treatment adherence in patients with gout. There is also a lack of randomized controlled trials that establish safe doses of acute and long-term treatment for gout, particularly in patients with IHD and stage 4 CKD and above (including end-stage renal failure). Furthermore, there is also a lack of studies showing optimal serum uric acid (SUA) target and validated clinical outcome measures, including disease activity and remission criteria for gout tailored to treat-to-target approaches and the high cost of newer gout medications. The causal relationship between asymptomatic hyperuricemia or gout with comorbidities such as IHD and CKD has yet to be fully elucidated. There is a pressing need for collaborative international efforts to address the overall suboptimal management of gout.

## Introduction and background

Introduction

Gout is a crystal-induced inflammatory arthropathy that is widely prevalent and has long been overlooked in terms of clinical significance, despite its potentially debilitating nature with a negative impact on the quality of life and cardiovascular health of those who suffer from it. It is one of the most frequent indications for referral to rheumatology clinics. Despite its prevalence and numerous international guidelines on the management of gout, many unmet needs remain. This review explores and addresses some of these unmet needs. While our focus is on the Singaporean population, we believe that many of the issues raised will also be relevant to the greater global population.

Background

Epidemiology in Singapore

Gout affects between <1% and 6% of the population globally, and the prevalence and incidence are increasing [1]. A local study [2] investigating a large cohort of 52,332 Chinese men and women aged 45-74 years found a prevalence of 4.1% in Singapore Chinese. Further, the prevalence of gout reported in this study may be underestimated in the Singapore population for several reasons. First, other ethnicities, such as Malay and Indians, were not included in the study. However, the Malay and Indians accounted for 13.5% and 9% of the population, respectively. Moreover, patients who presented with gout at a young age (before the age of 35) were mostly excluded based on the age cut-off (minimum age of 45 years), and though not explicitly stated, the majority of patients had gout for less than 10 years. Thus, patients who were not aware of their diagnosis were also excluded. Consequently, the actual prevalence of gout in Singapore remains to be determined.

In the same study, it was found that patients with gout had a higher risk of death from coronary heart disease (CHD) (hazard ratio [HR]: 1.38, 95% confidence interval [CI]: 1.10-1.73) [2]. A systematic review and meta-analysis examined the prevalence of different types of cardiovascular diseases in about one million gout patients. Hypertension had the highest prevalence of 63.9%, followed by heart failure (8.7%), cerebral vascular event (4.3%), and myocardial infarction (MI) (2.8%), indicating an overall increase in cardiovascular risk burden in patients with gout [3].

Hyperuricemia, and consequently gout, has been associated with low glomerular filtration rates (GFRs), CKD, and its progression [4]. In a meta-analysis that looked into the prevalence and risk of CKD and nephrolithiasis in gout patients, the pooled prevalence of CKD stage ≥3 or more and nephrolithiasis in gout patients was 24% and 14%, respectively. It was also found that patients with gout were more than twice as likely to have CKD stage ≥3 compared to those without gout [5]. This is also consistent with the data shown in a more recent UK study with a large sample size of more than 600,000 patients, which showed a 29% higher risk of gout patients progressing into advanced CKD compared to those without gout [6], including end-stage renal failure (ESRF) [7]. In Singapore, patients with gout had a higher risk of death from CKD (HR: 5.81, 95% CI: 3.61-9.37) [2], and they had concomitant comorbidities of CKD (32.3%) and ischemic heart disease (IHD) (20.2%) [8].

## Review

Impact of hyperuricemia and gout on ischemic heart disease (IHD) and chronic kidney disease (CKD)

Hyperuricemia and Gout with IHD

Uric acid (UA) metabolism is regulated by xanthine oxidoreductase, which converts hypoxanthine to xanthine and then to UA. UA is further degraded to allantoin by uricase, which is then further degraded into ammonia by urease. However, uricase is absent in humans, thus resulting in higher UA levels in vivo. Several putative mechanisms for the relationship between UA and cardiovascular diseases (CVDs) have been described. They include endothelial dysfunction, resulting in impaired secretion and loss of balance between vasodilatory agents, such as nitric oxide, prostaglandin I2 (prostacyclin), and vasoconstrictors (e.g., endothelin-1, thromboxane A2, and angiotensin II). The production of reactive oxygen species (ROS), particularly peroxynitrite, during excessive UA synthesis causes DNA damage, cell death, and lipid peroxidation. These conditions cause the progression of atherosclerosis [9]. A meta-analysis of 14 prospective observational studies with more than 300,000 patients further highlighted the increased risk of CVD secondary to high UA. The results from these studies indicate that for each increase of 1 mg/dl of serum uric acid (SUA), the overall risks of IHD and all-cause mortality increased by 20% and 9%, respectively [10]. The Coronary Artery Risk Development in Young Adults (CARDIA) study showed that an increasing SUA trajectory during young adulthood was associated with increased CVD incidence by middle age [11]. There is also an increased risk of mortality related to CVD (HR 1.29, 95% CI: 1.14-1.44) [12]. This is particularly true for patients with severe gout, characterized by elevated SUA concentrations (>0.55 mmol/L [>9.1 mg/dL]), longer disease duration (≥2 years), oligoarticular or polyarticular disease, and joint damage and tophi [13]. The 2018 European Society of Cardiology and European Society of Hypertension guidelines recommended screening for SUA as part of a risk stratification strategy [14].

As the prevalence of both IHD and CKD is high in patients with gout, it is essential to determine the added risk of one condition to another. One meta-analysis examining 27,000 patients with CKD found that every 1 mg/dl increase in SUA levels led to a 12% increased risk of cardiovascular mortality [15]. In their 2016 guidelines on managing gout, the European Alliance of Associations for Rheumatology (EULAR) recommended that patients with gout should be systematically screened for associated comorbidities and cardiovascular risk factors, given the high prevalence of cardiovascular comorbidities in gout patients, although the level of evidence to support the effectiveness of such an intervention on SUA is low [16]. The 2020 guidelines of the American College of Rheumatology (ACR) for gout [17] included conditional recommendations to reduce alcohol intake, adopt a low-purine diet, and prioritize weight loss. However, the screening of cardiovascular risk factors was not explicitly mentioned or recommended. Meanwhile, a summary of two Cochrane systematic reviews that examined the efficacy and safety of lifestyle interventions for treating gout [18] and a meta-analysis [19] that tested the relationship between different dietary components (both high- and low-purine diets) and inherited genetic variants with SUA levels showed little variation in SUA levels in the general population. Although gout is associated with an increased prevalence of cardiovascular morbidity and mortality, there is a scarcity of high levels of evidence on active screening, representing another unmet need in gout management.

Gout With CKD

Similarly, in CKD, endothelial dysfunction, activation of the renin-angiotensin-aldosterone system, and oxidative stress are some of the proposed mechanisms through which UA affects the progression of CKD [20]. Whether UA is a causal factor inducing inflammation, oxidative stress, and endothelial dysfunction per se has not been determined. One study used Mendelian randomization (MR) to evaluate the causal effect of high SUA on CKD. Over 100,000 patients were involved, and 26 urate-associated single-nucleotide variants (SNVs) were identified. However, the result did not support a causal association between SUA and the presence of CKD or GFR [21]. However, in a meta-analysis and systemic review, a 1 mg/dl increase in SUA levels was associated with an 8% increased mortality risk [22]. Thus, it is essential to treat gout in patients with CKD. Unfortunately, the management of gout in this group of patients remains challenging due to the increased risk of side effects from drugs, such as colchicine, nonsteroidal anti-inflammatory drugs (NSAIDs), and allopurinol, the ineffectiveness of uricosuric agents, and the lack of evidence to support the use of febuxostat and pegloticase in advanced CKD [23]. This will be further elaborated on in the following section.

Treatment of acute gout attacks

Colchicine, nonsteroidal anti-inflammatory drugs (NSAIDs), and oral or intra-articular glucocorticoids (GC), whether used alone or in combination, are recommended during an acute gout flare, with no prioritization between the agents due to the lack of comparative evidence [16,17]. Therefore, the choice of acute agents often depends on the physician's preference after considering the patient's comorbidities, allergy history, and side effect profile. Anecdotally, patients' preference for infections over oral medications (or vice versa) and previous experiences often influence physicians' decisions when making joint therapeutic decisions in the real-world setting. There are, at present, no studies to substantiate this.

Colchicine

The AGREE (Acute Gout Flare Receiving Colchicine Evaluation) trial showed that low-dose colchicine is as productive as a high-dose regime, achieving >50% pain reduction in patients with acute gout flare, with a safety profile similar to placebo [24]. Concerns with colchicine used in patients with impaired kidney function include neuromyopathy and rhabdomyolysis, particularly in the case of drug-drug interaction with statins, cyclosporin, and clarithromycin as this group of patients tends to have more comorbidities requiring more medications [25]. This is likely due to poor colchicine clearance, particularly in those with eGFR of <30 ml/min/1.73 m^2^ Dialysis clearance of colchicine is poor as observed in a pharmacokinetic study [26]. There is a scarcity of randomized controlled trials (RCTs) examining colchicine use in patients with impaired kidney function, and most existing studies have varying dosing and frequency patterns [25]. Moreover, another systemic review showed that there were occasions when patients with risk factors or contraindications for colchicine were still prescribed it during gout flare [27]. This illustrates knowledge gaps for healthcare providers when treating gout, highlighting an unmet need for evidence to support safe renally adjusted colchicine dosing and selection criteria for gout patients with kidney impairment.

Nonsteroidal Anti-inflammatory Drugs (NSAIDs)

NSAIDs are an effective treatment for gout flare but are associated with adverse effects, such as acute and chronic renal failure, nephrotic syndrome, and tubulointerstitial nephritis (TIN) [28]. The mechanism of this effect is associated with the inhibition of prostanoids, such as prostaglandin E2 (PGE2) and prostacyclin (PGI2), resulting in poor renal perfusion. The inhibition of cyclooxygenases, such as COX-1 and COX2, by NSAIDs also results in high blood pressure and sodium retention. Moreover, TIN can be caused by glomerular basement membrane damage, reduction in pore size, and podocyte density due to the use of NSAIDs [29]. A meta-analysis found that cyclooxygenase-2 (COX-2) inhibitors and traditional NSAIDs, except naproxen, increased the risk of serious CV events and death [30]. In the PRECISION (Prospective Randomized Evaluation of Celecoxib Integrated Safety versus Ibuprofen or Naproxen) trial, celecoxib, when given in a moderate dose, was found to have a similar cardiovascular risk to naproxen, suggesting a dose and risk association [31]. These issues further limit the treatment choices for gout flares, especially for gout patients with kidney impairment, who also have increased cardiovascular risk factors.

Prednisolone

Prednisolone is an effective treatment for gout flare [32,33], particularly when colchicine and NSAIDs are contraindicated due to kidney impairment. However, GC use should also be carefully contemplated in the presence of comorbidities such as diabetes, hypertension, and infection, especially in patients with end-stage renal disease (ESRD), as it can exacerbate these conditions.

Anti-interleukin 1 Agents

Anti-interleukin 1 (IL-1) receptor antagonists, such as anakinra [34-36], effectively relieve gout flare quickly. Currently, there is no high-level evidence of their use in cases of advanced CKD. Canakinumab, an anti-IL1B monoclonal antibody, effectively treats gout flares [37]. However, the costs and availability of both these agents in Singapore limit their use.

The long-term management of gout

Evidence to Support the Initiation of Urate-Lowering Therapy (ULT)

ULT is the key to controlling and curing gout. As described previously, high SUA levels are associated with cardiac comorbidities. The recently published ALL-HEART (Allopurinol Versus Usual Care in UK Patients With Ischemic Heart Disease) trial [38] investigated whether allopurinol therapy improves major cardiovascular outcomes (composite of nonfatal MI, nonfatal stroke, or cardiovascular death) in patients with IHD and asymptomatic hyperuricemia and found that allopurinol failed to improve major cardiovascular outcomes. Another study also failed to demonstrate a delay in carotid atherosclerosis progression in patients with asymptomatic hyperuricemia started on febuxostat [39].

Nevertheless, ULT commencement is essential in patients with gout and cardiovascular risk factors to reduce cardiovascular risks and gout flares. A recent retrospective observational study in the United Kingdom included 62,574 patients in a multivariable nested case-control study to investigate whether there was a transient increase in the risk of cardiovascular events (acute MI or stroke) after a recent gout flare. The results showed that patients with cardiovascular risk factors were more likely to develop gout flares, especially in the first 60 and 120 days, with odds ratios (ORs) of 1.93 [95% CI: 1.57-2.38]) and 1.06 [95% CI: 0.84-1.34]), respectively. Similarly, patients with gout flare also had significantly higher cardiovascular event rates per 1000 person-days of 2.49 (95% CI: 2.16-2.82) and 2.16 (95% CI: 1.85-2.47), respectively, from day 60 up to day 120. This highlights the importance of ULT in patients with gout to reduce cardiovascular risk [40]. While allopurinol does not significantly reduce major adverse cardiac events (MACE) and mortality, studies have demonstrated its blood pressure-lowering properties, possibly through a direct reduction of xanthine oxidase-mediated oxidative stress, indicating the critical role of ULT in treating patients with coronary artery disease (CAD) with or without gout [41]. Febuxostat was also found to have a reno-protective effect and lower rates of microalbuminuria, progression to overt proteinuria, or worsening of overt proteinuria to ≥300 mg/g creatinine [42,43]. ULT is recommended if there are two or more flares per year, tophi and urate arthropathy, or renal calculi [16,17]. Based on a systemic review and meta-analysis, moderate-quality evidence shows that initiating ULT during gout flare does not increase pain severity, the risk of ULT discontinuation [44], or flare duration [45]. However, the initiation rate of ULT remains low. The factors affecting ULT use will be explained in the following sections.

Treat-to-Target Strategy

Monosodium urate (MSU) crystals form when serum concentrations reach >6.0 mg/dl (>0.36 mmol/l), typically in the peripheral joints, with a lower-than-core body temperature of 35°C [46]. MSU crystals then activate monocytes and macrophages, resulting in the NLRP3 (NLR family pyrin domain containing 3) inflammasome-mediated release of IL-1B and proinflammatory cytokines and recruitment of neutrophils to the site of crystal deposition, driving the clinical features of gout [47]. The treat-to-target strategy has gained popularity in the management of many rheumatological conditions. Major rheumatological societies advocate using treat-to-target SUA strategies as part of the long-term management of gout. SUA levels of <6 mg/dl (<0.36 mmol/l) [16,17] and 5 mg/dl (<0.30 mmol/l) are recommended, with the latter target being for patients with tophaceous gout [16].

In multiple studies, when gout patients were treated with an SUA target level of <6 mg/dl (<0.36 mmol/l), the MSU crystal load in joints visualized with both ultrasound and dual-energy computed tomography (DECT) was found to have reduced significantly [48-50]. It was observed that the baseline radiological MSU crystal burden predicted a flare rate of up to two years, supporting the use of ULT early in the disease course [50,51]. Tophi regression and slowing of joint damage progression were also observed in patients who responded to ULT to achieve an SUA level <6 mg/dl (<0.36 mmol/l) [50,52]. The findings from the Italian Uric Acid Right for Heart Health (URRAH) study showed the cardiovascular implications of SUA. Specifically, SUA was identified as an independent factor associated with total and cardiovascular deaths via multivariate analyses (HR = 2.08, 95% CI: 1.146-2.97; P < 0.001), which further established the association between SUA levels and cardiovascular risk. However, the SUA level that was found to predict cardiovascular mortality according to the heart score risk chart was 5.6 mg/d [53]. A more intensive ULT regime targeting an SUA level of <3.4 mg/dl (<0.20 mmol/l) showed similar clinical benefits according to the Outcome Measures in Rheumatology (OMERACT) core outcome domains (gout flare, tophi, pain, patient's global assessment of disease activity, health-related quality of life, and activity limitation) compared to patients with a target SUA of <5.0 mg/dl (<0.30 mmol/l) [54]. The benefits of achieving lower SUA targets are still unclear, apart from a potential neuroprotective effect seen in observational studies [55-57]. Further, there has been a lack of focus on composite clinical outcome measures in all the major gout trials. These issues highlight the gaps in gout management and warrant more research to identify the best SUA targets and validated clinical outcome measures, including measuring disease activities and remission criteria for gout tailored to the treat-to-target approach [58].

Xanthine Oxidase Inhibitors (XOI)

Hande et al. recommended allopurinol dosing based on creatinine clearance [59]. ACR 2021 recommended starting allopurinol lower (≤100 mg/day and febuxostat (≤40 mg/day) in patients with CKD (stage ≥3) [17]. The CARES (Cardiovascular Safety of Febuxostat and Allopurinol in Patients with Gout and Cardiovascular Morbidities) trial [60] demonstrated increased cardiac-related mortality with the use of febuxostat; however, this was not seen in the PRIZE (Program of Vascular Evaluation Under Uric Acid Control by Xanthine Oxidase Inhibitor, Febuxostat) [39], FREED (Febuxostat for Cerebral and Cardiorenovascular Events Prevention Study) [43], FEATHER (Febuxostat Versus Placebo Randomized Controlled Trial Regarding Reduced Renal Function in Patients With Hyperuricemia Complicated by Chronic Kidney Disease Stage 3) [61], or FAST (long-term cardiovascular safety of febuxostat compared with allopurinol in patients with gout) [62] trials. Even so, the United States Food and Drug Administration (FDA) has yet to lift the black box warning of increased cardiac-related death using febuxostat. This may continue to cause hesitance in doctors to prescribe febuxostat in patients with IHD. Similarly, while UA levels predict the progression of CKD, in three systematic reviews and meta-analyses reviewing the effect of ULT on renal outcomes in CKD patients, ULT resulted in modest improvements in creatinine and eGFR and slowed the progression of CKD. Most studies included were small, single-center, observational cohort studies with short durations or follow-up periods [63-65]. More recent RCTs, such as the FEATHER [61], PERL (serum urate lowering with allopurinol and kidney function in type 1 diabetes) [66], and CKD-FIX (Controlled trial of slowing of Kidney Disease progression From the Inhibition of Xanthine oxidase) [67] trials, did not show clinically meaningful benefits with SUA reduction using allopurinol and febuxostat in patients with CKD up to stage 4 without gout. Further, a Taiwanese paper showed that allopurinol and febuxostat are associated with cutaneous adverse reactions (CARs), including fatal CAR, although the frequency was higher in the allopurinol group [68]. However, another study showed that the hypersensitivity reaction (HSR) rate did not differ significantly between allopurinol and febuxostat use [69]. Risk factors of CAR include a high initiation dose of allopurinol [68,70] and poorer kidney function [70,71], especially when HLA-B5801 is present, although these were not observed in a febuxostat group [68]. Allopurinol and febuxostat have also been associated with acute kidney failure. A post-marketing study reported that the risk of acute renal failure (ARF) was 5.7 and 3.3 times more frequent with febuxostat and allopurinol, respectively, than with other drugs, such as NSAIDs, diuretics, antibiotics, and immunosuppressive therapies [72]. In a study that enrolled 96 people with an eGFR range of 15-50 ml/min/1.73 m^2^ (37.5% were stage 4 CKD patients), febuxostat use up to 80 mg daily did not result in a decline in renal function [73]. In many other studies involving febuxostat, such as the PRIZE [39], FREED [43], CARES [60], FEATHER [61], and FAST [62] trials, patients with CKD 4 and above were excluded. Thus, there is a need to study the efficacy, optimal dose, and safety of febuxostat in patients with CKD 4 and above, especially when allopurinol is contraindicated. This is of paramount importance for Singapore as most of the population is descended from Han Chinese, with whom the HLA-B5801 association with severe cutaneous adverse reactions (SCAR) is well established.

Uricosuric Agents

Uricosuric is recommended when there is a contraindication or lack of treatment response to XOI [16,17]. However, uricosuric agent use is limited in renally impaired patients. Further, uricosuric agents require adequate hydration, which is detrimental to patients with poor cardiac and renal function, to prevent UA nephrolithiasis. Probenecid is contraindicated in patients with eGFR < 50 ml/minute, urate nephrolithiasis, and drug-drug interaction. Lesinurad and lesinurad plus allopurinol combination tablets were withdrawn from the US market. These drugs were expensive and included a black box warning of acute kidney failure from the FDA [74]. Benzbromarone is a potent ULT comparable to allopurinol, and its efficacy in reducing gout flares and tophi reduction has been established in multiple studies [75-77]. It can be used in patients with eGFR of >30 ml/minute. However, benzbromarone was withdrawn from the European market due to hepatotoxicity concerns [78]. In Singapore, benzbromarone is approved for use as a uricosuric agent.

Uricases

The lack of uricase in humans results in the inability to convert UA to allantoin, a water-soluble molecule readily excreted by the kidneys, leading to UA accumulation. Pegloticase, a polyethylene glycol (PEG) uricase, helps reduce SUA levels [79] and tophi burden in patients with chronic severe gout who are refractory or intolerant to conventional ULT [52]. Evidence regarding the long-term use of pegloticase is lacking. Infusion reactions, including anaphylaxis, are attributed to the development of anti-drug antibodies (ADA) and are the most common side effect of pegloticase use, which render pegloticase ineffective [52,79,80]. Because of the presence of ADA, the concomitant use of pegloticase with methotrexate (MTX) was studied, and an improved pegloticase response rate was observed in the case series [81,82]. In the recent EULAR 2022 congress, the MIRROR (Pegloticase in Combination With Methotrexate in Patients With Uncontrolled Gout) trial showed a marked pegloticase response rate at month 6 when pegloticase was used with MTX compared to pegloticase monotherapy [83]. This suggests more applicability and may lead to confidence in pegloticase use in the future. However, the high cost and lack of availability limit the usage of pegloticase in Singapore.

Barriers to the initiation, escalation, and adherence to treatment

In the United Kingdom, Australia, and China, it was found that non-rheumatologists, general practitioners (GPs), and primary care nurses were frequently unaware or poorly versed in the management principles reflected in the gout treatment guidelines [84-86]. This lack of knowledge led to doctors' reluctance to initiate ULT, and patients with gout had low adherence to and continuation of ULT [87]. The initiation rate of ULT in gout patients is low. Only 20%-30% of patients in the United Kingdom were prescribed ULT [88,89]. Some of the physician-related factors identified were a lack of information about the safety of ULT, the need to continue ULT during acute attacks, and the need for long-term ULT to cure gout [84] potentially. A Singapore study revealed that while 67% of patients in the cohort were started on ULT, only 22% achieved SUA targets. This study also found that patients with two or more comorbidities had higher odds of attaining the ULT target. This signals that regular healthcare reviews may positively influence health behaviors and ensure prescription refills, and that optimal gout management is possible even in multiple comorbidities [8,90].

In multiple international studies, the adherence rate of patients to ULT was only found to reach 47% [88,91-93]. Further, the 12-month persistent adherence rate for this group of patients was even lower at 28% [88,92,94]. A lack of counseling by care providers, poor understanding of potential gout flares during the initiation of ULT, and insufficient awareness of ULT as a long-term treatment for gout were some of the reasons for poor adherence observed in the United States and China [95,96]. Another factor that may limit allopurinol use is the concern of developing a severe CAR (SCAR). In a local study on hospitalized patients with drug allergies, allopurinol was the culprit in 5.7% of patients. It accounted for 38% of cases of drug rash with eosinophilia and systemic symptoms (DRESS). Interventions are available to address and improve the issues described above. A study in the United States involving 665 primary care physicians found that didactic lectures on gout could improve the physicians' knowledge and change their practice in line with clinical guidelines, including knowing target SUA levels, when to initiate ULT, and how to manage acute and chronic gout [97]. Education of patients by healthcare providers is an effective strategy for motivating patients to adhere to ULT [87,98]. Optimal treatment of gout is achievable with a multidisciplinary approach focused on improving physicians' prescription habits and increasing patients' education and empowerment. A local study showed that following this approach, the percentage of gout patients achieving target SUA levels improved from 27% to 72% in one year [99]. Education, through a predominantly nurse-led intervention, can improve adherence dramatically, with 92% of patients achieving their target levels in one study [100]. Locally, there are also pharmacist-led gout clinics and nurse-led interventions to help address patients' concerns related to gout treatment.

Another barrier to gout treatment in Singapore is the cost of medications. Expensive medications are funded through a complex mix of personal out-of-pocket payment, hospitalization-focused personal and government-aided medical insurance schemes, and limited government subsidies, which are only available to patients in lower income classes. The Medication Assistance Fund (MAF) was established in 2013 to support the prescription of expensive drugs for specific clinical indications. Another scheme, the MAF Plus scheme, was launched for patients taking high-cost drugs that are not on the standard drug list (SDL) or MAF list. Costly gout treatments, such as anakinra, pegloticase, febuxostat, and benzbromarone, do not fall under the SDL and MAF and can thus only be considered for the MAF Plus scheme on a case-by-case basis. While these supports exist for patients who cannot afford certain medications, the application process can be tedious. The need to produce supporting documents, for example, from working children even if they do not live with the involved patients and patients' unwillingness to burden their children are some factors that may deter patients from applying for this aid.

## Conclusions

The continuous increase in the prevalence and incidence of gout globally is worrisome as it contributes to the increasing prevalence and incidence of associated comorbidities. This is further exacerbated by poor ULT uptake, adherence, and persistence. The review identified the following unmet needs in gout management: an accurate and up-to-date prevalence of gout locally, the need for more high-level evidence that supports active screening of SUA in patients with CVD, the need for RCTs to establish safe doses of acute and long-term treatment for gout, particularly in patients with IHD and stage 4 CKD and above (including ESRD), measures to improve the initiation rate of ULT by physicians and patient adherence to treatment, and, last but not least, the need for studies that show optimal SUA targets and validated clinical outcome measures (including the measurement of disease activities and remission criteria for treat-to-target approaches in the management of gout). Collaborative international efforts are crucial for improving the widespread suboptimal management of gout.
